# Optimizing reaction paths for methanol synthesis from CO_2_ hydrogenation via metal-ligand cooperativity

**DOI:** 10.1038/s41467-019-09918-z

**Published:** 2019-04-23

**Authors:** Yizhen Chen, Hongliang Li, Wanghui Zhao, Wenbo Zhang, Jiawei Li, Wei Li, Xusheng Zheng, Wensheng Yan, Wenhua Zhang, Junfa Zhu, Rui Si, Jie Zeng

**Affiliations:** 1 Hefei National Laboratory for Physical Sciences at the Microscale, Key Laboratory of Strongly-Coupled Quantum Matter Physics of Chinese Academy of Sciences, National Synchrotron Radiation Laboratory, Key Laboratory of Surface and Interface Chemistry and Energy Catalysis of Anhui Higher Education Institutes, Department of Chemical Physics, University of Science and Technology of China, 230026 Hefei, Anhui People’s Republic of China; 2 Shanghai Synchrotron Radiation Facility, Shanghai Institute of Applied Physics, Chinese Academy of Sciences, 201204 Shanghai, People’s Republic of China

**Keywords:** Catalyst synthesis, Catalytic mechanisms, Heterogeneous catalysis

## Abstract

As diversified reaction paths exist over practical catalysts towards CO_2_ hydrogenation, it is highly desiderated to precisely control the reaction path for developing efficient catalysts. Herein, we report that the ensemble of Pt single atoms coordinated with oxygen atoms in MIL-101 (Pt_1_@MIL) induces distinct reaction path to improve selective hydrogenation of CO_2_ into methanol. Pt_1_@MIL achieves the turnover frequency number of 117 h^−1^ in DMF under 32 bar at 150 °C, which is 5.6 times that of Pt_n_@MIL. Moreover, the selectivity for methanol is 90.3% over Pt_1_@MIL, much higher than that (13.3%) over Pt_n_@MIL with CO as the major product. According to mechanistic studies, CO_2_ is hydrogenated into HCOO* as the intermediate for Pt_1_@MIL, whereas COOH* serves as the intermediate for Pt_n_@MIL. The unique reaction path over Pt_1_@MIL not only lowers the activation energy for the enhanced catalytic activity, but also contributes to the high selectivity for methanol.

## Abstract (web summary)

Controlling the reaction path is instrumental for developing efficient catalysts for CO_2_ hydrogenation. Here, the authors report that the ensemble of Pt single atoms coordinated with oxygen atoms in MIL-101 induces distinct reaction path to improve selective hydrogenation of CO_2_ into methanol relative to nanocrystal counterparts.

## Introduction

Hydrogenation of CO_2_ into fuels and useful chemicals serves as an important process which helps to alleviate the dearth of fossil fuels^1–17^. According to both experimental and theoretical studies, CO_2_ hydrogenation involves various reaction paths^4–6^. Even for the first step to activate CO_2_, CO_2_ can be decomposed into CO*, transformed into carboxyl intermediate (COOH*), or hydrogenated into formate intermediate (HCOO*)^6–9^. Over practical catalysts, diversified reaction paths inevitably coexist, which induces the formation of different products such as CO, methane, formic acid, methanol, higher alcohols, and even gasoline, generally limiting the selectivity for the target product^10–14^. For instance, Cu/ZnO/Al_2_O_3_ catalysts have already been applied to realize gas-phase CO_2_ hydrogenation into methanol in industry, but suffer from the limited selectivity (<70%) for methanol and stringent reaction conditions (50–100 bar, 200–300 °C)^15,16^. Catalyzed by Cu/ZnO/Al_2_O_3_, CO_2_ undergoes reverse water-gas shift path (RWGS) to produce CO* via the formation of COOH* species^17^. Since such catalysts also involve high energy barrier for the transformation of CO*, a large proportion of CO* is directly desorbed from the catalyst surfaces to form gaseous CO, competing over further hydrogenation into methanol^17^. Recently, constructing highly active interfaces such as Cu/CeO_x_, Cu/ZnO, and Cu/ZrO_2_ interfaces has been reported to efficiently lower the energy barrier for the transformation of CO*, which not only increases the selectivity for methanol but also elevates the catalytic activity^18–23^. In addition, another pivotal approach is based on engineering the coordination environment of active metal atoms by alloying^24,25^. For instance, the fabrication of NiGa catalysts was found to suppress the RWGS path, thereby facilitating the production of methanol^26^. Co_4_N nanosheets were reconstructed into Co_4_NH_x_ during aqueous CO_2_ hydrogenation into methanol, wherein the amido-hydrogen atoms directly added CO_2_ to form HCOO*^4^^,^. Despite of these achievements, it is still highly desiderated to efficiently control the reaction path for developing highly active and selective catalysts toward CO_2_ hydrogenation.

Herein, we demonstrate that metal-ligand cooperativity in Pt single atoms encapsulated in MIL-101 (Pt_1_@MIL) varies the reaction path and improves the selective hydrogenation of CO_2_ into methanol relative to nanocrystal counterparts (Pt_n_@MIL). In Pt_1_@MIL, every Pt single atom and its coordinated O atoms compose an active center. During CO_2_ hydrogenation, the turnover frequency (TOF) number of Pt_1_@MIL reaches 117 h^−1^ in DMF under 32 bar at 150 °C, being 5.6 times as high as that (21 h^−1^) of Pt_n_@MIL. Moreover, the selectivity for methanol reaches 90.3% over Pt_1_@MIL, whereas the major product for Pt_n_@MIL is CO with the selectivity of 57.5%. The cooperativity between Pt single atoms and their coordinated O atoms in Pt_1_@MIL enables the dissociation of H_2_ to form O–H groups. The hydroxy H atoms add into CO_2_ to produce HCOO* as the intermediates. As for Pt_n_@MIL, Pt–H is formed, wherein H atoms hydrogenate CO_2_ into COOH* as the intermediates. The unique reaction path over Pt_1_@MIL not only lowers the activation energy for the enhanced catalytic activity, but also contributes to the high selectivity for methanol.

## Results

### Synthesis and structural characterizations of Pt_1_@MIL

**Fig. 1:**
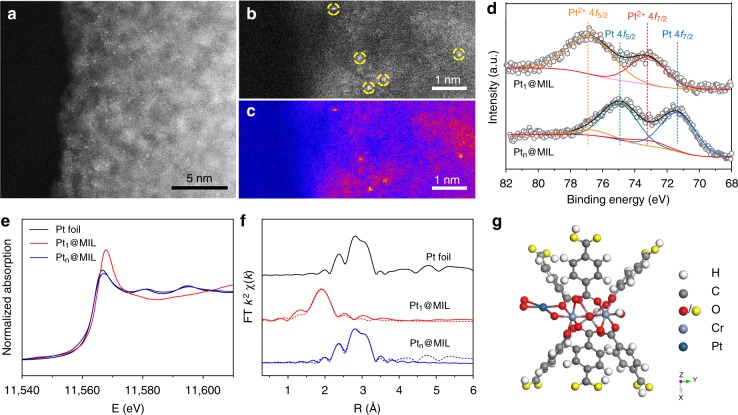
Structural characterizations of Pt@MIL. **a** HAADF-STEM image of Pt_1_@MIL. **b**, **c** Magnified HAADF-STEM image and its corresponding color-coded intensity map of Pt_1_@MIL. Pt single atoms marked in yellow circles were uniformly anchored in MIL-101. **d** XPS spectra of Pt 4 *f* for Pt_1_@MIL and Pt_n_@MIL. **e** Pt L_3_-edge XANES profiles of Pt foil, Pt_1_@MIL, and Pt_n_@MIL. **f** Pt L_3_-edge EXAFS spectra in *R* space of Pt foil, Pt_1_@MIL, and Pt_n_@MIL. Pt foil was used as the reference. **g** Structural model of Pt_1_@MIL simulated by DFT calculations. Blue, violet, red, gray, and white spheres represent for Pt, Cr, O, C, and H atoms, respectively. The yellow spheres represent the oxygen atoms which are fixed at their positions in crystal

MIL-101 is a typical MOF which consists of μ_3_-oxo bridged Cr(III)-trimers cross linked by terephthalic acid^27,28^. The high specific surface area of S_Langmuir_ = 5900 m^2^ g^−1^ and large pore volume of ca. 2.0 cm^3^ g^−1^ render MIL-101 high adsorption capacities, making it an attractive candidate for gas adsorption. As such, MIL-101 with an average particle size of 500 nm was synthesized based on previous literatures^27,28^ (Supplementary Fig. 1), serving as the support in this work. In a typical synthesis of Pt_1_@MIL, K_2_PtCl_4_ and NaBH_4_ aqueous solutions were added into the flask containing MIL-101 aqueous solution via a syringe pump under magnetic stirring. In Pt_1_@MIL, the Pt mass loading was determined as 0.2% by inductively coupled plasma–atomic emission spectroscopy (ICP–AES). Figure 1a shows a high-angle annular dark-field scanning transmission electron microscopy (HAADF–STEM) image of Pt_1_@MIL. As manifested by brightness, Pt atoms were atomically dispersed in MIL-101 in the absence of nanoparticles. To highlight the dopant atoms, the magnified HAADF–STEM image and its corresponding color-coded intensity map were shown in Fig. 1b, c, indicating the isolated distribution of Pt atoms. After Pt_1_@MIL was further washed for different rounds, the ratio of Pt to Cr remained almost unchanged, confirming that Pt single atoms were indeed anchored in MIL-101, rather than serving as a residual (Supplementary Fig. 2). By simply increasing the concentrations of K_2_PtCl_4_ and NaBH_4_ aqueous solutions, Pt_n_@MIL with the Pt mass loading of 1.0% was facilely synthesized. As shown in the HAADF–STEM image of Pt_n_@MIL, Pt nanocrystals with an average size of 1.8 nm were observed (Supplementary Fig. 3). The ratio of surface Pt atoms in Pt_n_@MIL was determined as 41.5% by CO pulse chemisorptions (Supplementary Fig. 4). To characterize the electronic properties of the obtained samples, we conducted X-ray photoelectron spectroscopy (XPS) measurements of Pt_1_@MIL and Pt_n_@MIL. As shown in Fig. 1d, the Pt species in Pt_1_@MIL was determined to be at Pt^2+^ state. By comparison, most of Pt species in Pt_n_@MIL was at the metallic state, whereas a small portion of Pt was oxidized.

The X-ray absorption near-edge spectroscopy (XANES) and extended X-ray absorption fine structure (EXAFS) were measured to determine the electronic and coordination structures of Pt atoms in Pt@MIL catalysts. The Pt L_3_-edge XANES profiles in Fig. 1e indicate that the Pt species in Pt_1_@MIL were in a higher oxidation state than those in Pt_n_@MIL, according to the stronger intensity for white line. It is worth noting that the spectrum of Pt_n_@MIL was similar to that of Pt foil, indicating the metallic state of Pt species in Pt_n_@MIL, consistent with the XPS results (Fig. 1d). As shown in EXAFS in *R* space (Fig. 1f), Pt_1_@MIL exhibited a prominent peak at 2.01 Å from the Pt–O shell with a coordination number (*CN*) of 3.7 (Supplementary Table 1). No other typical peaks for Pt–Pt contribution at longer distances (>2.5 Å) were observed, revealing the isolated dispersion of Pt atoms throughout the whole Pt_1_@MIL. As for Pt_n_@MIL, a new peak at 2.76 Å was observed, corresponding to the Pt–Pt metallic bond with a *CN* of 7.4.

To investigate the location and coordination of Pt single atoms in MIL-101, density functional theory (DFT) calculations were performed to establish the atomic model of Pt_1_@MIL. The building unit of MIL-101 comprises terephthalic linkers and an inorganic trimer. The trimer consists of three Cr atoms in an octahedral coordination. The vertices of octahedral coordination around each Cr atom are occupied by four O atoms from carboxylate groups in terephthalic linkers, one μ_3_O atom in the middle of Cr trimers, and one O atom from the terminal water or fluorine group. Both XPS and XANES results indicate an oxidation state assignment of Pt^2+^ for Pt_1_@MIL (Fig. 1d, e). In Pt_1_@MIL, the individual Pt atom was stabilized by O atoms in a planar four-coordinate geometry with one O atom in carboxyl, one dangling O connected with a Cr atom, and one O_2_ moiety (Fig. 1g and Supplementary Fig. 5). This optimized model is consistent with the oxidation state of Pt^2+^ indicated by XPS and XANES results, because Pt^2+^ generally adopts the *dsp*^2^ hybridization which results in the planar four-coordinate geometry. To further confirm the Pt oxidation states in this model, we have compared the charge of the Pt atom in Pt_1_@MIL with that in isolated Pt–O and O–Pt–O clusters as representatives of Pt^2+^ and Pt^4+^ (Supplementary Fig. 6). Based on Mullinken analysis, the charges of Pt atoms in Pt–O and O–Pt–O clusters are +0.72 and +1.52 e, respectively. The Pt single atom in Pt_1_@MIL is + 0.59 e charged, approximating to that in Pt–O cluster. Thus, the oxidation state of Pt in Pt_1_@MIL was assigned to +2. Moreover, the model of the Pt single atom coordinated with four O atoms in Pt_1_@MIL matches our EXAFS result in terms of the *CN* of Pt–O.

### Catalytic properties of Pt_1_@MIL for CO_2_ hydrogenation

**Fig. 2:**
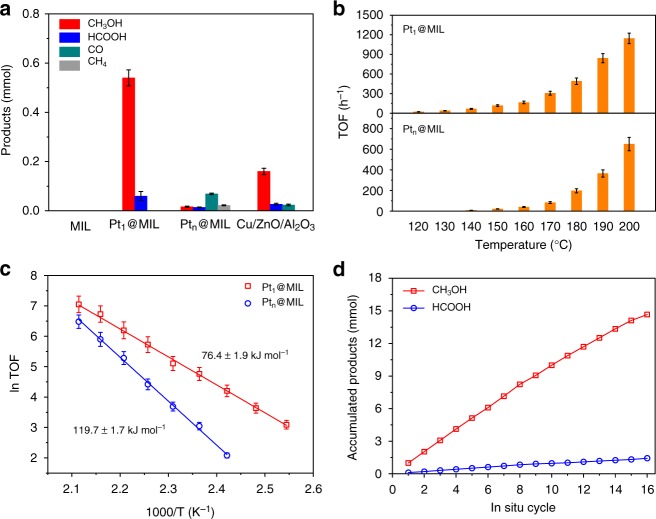
Catalytic performance of Pt@MIL in CO_2_ hydrogenation. **a** Comparison of products obtained by using MIL-101, Pt_1_@MIL, Pt_n_@MIL, and commercial Cu/ZnO/Al_2_O_3_ in DMF under CO_2_/H_2_ mixed gas (CO_2_:H_2_ = 1:3, 32 bar) at 150 °C after 1 h. **b** Comparison of TOF numbers of Pt_1_@MIL and Pt_n_@MIL in DMF under CO_2_/H_2_ mixed gas (CO_2_:H_2_ = 1:3, 32 bar) at different temperatures. **c** The Arrhenius plots of Pt_1_@MIL and Pt_n_@MIL. **d** Products obtained by conducting in-situ cycles over Pt_1_@MIL. For each cycle, the catalytic reaction proceeded under 32 bar of CO_2_/H_2_ mixed gas (CO_2_/H_2_ = 1:3) at 150 °C for 6 h. For each catalytic test, the amounts of Pt_1_@MIL and Pt_n_@MIL were controlled at 500 and 240 mg, respectively, to keep the same amount (1.0 mg) of exposed Pt atoms. Error bars represent standard deviation from three independent measurements

The catalytic properties of the as-obtained Pt@MIL in CO_2_ hydrogenation were evaluated in a slurry reactor with 10 mL of DMF under 32 bar of CO_2_/H_2_ mixed gas (CO_2_:H_2_ = 1:3) at 150 °C. As a benchmark, commercial Cu/ZnO/Al_2_O_3_ (63 wt% Cu) was directly purchased from Alfa Aesar, with the ratio of surface Cu atoms to total atoms determined as 32.5% (Supplementary Fig. 7). For each catalytic test, the amounts of Pt_1_@MIL and Pt_n_@MIL were controlled at 500 and 240 mg, respectively, to keep the same amount (1.0 mg) of exposed Pt atoms, whereas 20 mg of Cu/ZnO/Al_2_O_3_ was used. When the reaction was catalyzed by MIL-101, the product was below detection limit (Fig. 2a). As for Pt_1_@MIL, 0.6 mmol of products were generated after 1 h, whereas the product yield was 0.12 mmol for Pt_n_@MIL. More importantly, the selectivity for methanol reached 90.3% over Pt_1_@MIL with the production of formic acid as the by-product (Fig. 2a). With regard to Pt_n_@MIL, the major product was CO with the selectivity of 57.5%, while the selectivities for formic acid, methanol, and methane were 11.1%, 13.3%, and 18.1%, respectively (Fig. 2a). In general, Pt-based heterogeneous catalysts have been reported to be selective to CO or methane during CO_2_ hydrogenation^29,30^. In this case, Pt_1_@MIL did not give rise to the formation of gaseous products, behaving much differently from conventional Pt-based catalysts. As for Cu/ZnO/Al_2_O_3_, 0.16 mmol of methanol, 0.026 mmol of formic acid, and 0.024 mmol of CO were formed after 1 h (Fig. 2a and Supplementary Fig. 8). To compare the catalytic activity more accurately, we calculated the TOF numbers of these catalysts by only taking Pt atoms into account based on the reaction profile at the initial stage. The TOF number of Pt_1_@MIL reached 117 h^−1^ in DMF under 32 bar at 150 °C, being around 5.6 times that (21 h^−1^) of Pt_n_@MIL and 39 times that (3 h^−1^) of Cu/ZnO/Al_2_O_3_ (Fig. 2b). As a benchmark, the activity of Cu/ZnO/Al_2_O_3_ in DMF solvent was comparable with the values obtained in a fixed-bed reactor^26,31–33^. We have further applied deuterated DMF (C_3_D_7_NO) as the solvent to replace DMF in CO_2_ hydrogenation over Pt_1_@MIL under 32 bar at 150 °C. The TOF number of Pt_1_@MIL reached 107 h^−1^ in C_3_D_7_NO, almost equal to the corresponding values in DMF (Supplementary Fig. 9). Accordingly, the effect of proton transfer in DMF on CO_2_ hydrogenation over Pt_1_@MIL could be neglected.

To explore the differences in catalytic properties between Pt_1_@MIL and Pt_n_@MIL, we conducted a series of catalytic tests under 32 bar of CO_2_/H_2_ mixed gas (CO_2_:H_2_ = 1:3) at different temperatures. As shown in Fig. 2b, Pt_1_@MIL exhibited much higher catalytic activity than Pt_n_@MIL (Supplementary Figs. 10 and 11). Arrhenius plots were obtained based on the linear fitting of *ln*TOF vs. 1000/T (Fig. 2c). The activation energy for Pt_1_@MIL was 76.4 kJ mol^−1^, much lower than that (119.7 kJ mol^−1^) for Pt_n_@MIL. To investigate the size effect, Pt nanoparticles with an average size of 1.2 nm on MIL-101 were prepared and evaluated under 32 bar at 150 °C (Supplementary Fig. 12). The TOF number was calculated to be 22 h^−1^ which was lower than that (117 h^−1^) of Pt_1_@MIL and basically the same as that (21 h^−1^) of Pt_n_@MIL with 1.8-nm Pt nanoparticles (Supplementary Fig. 13). As such, Pt_1_@MIL exhibited higher catalytic activity and selectivity for methanol than Pt_n_@MIL even with tiny Pt clusters.

To explore stability of Pt_1_@MIL, we performed successive rounds of reaction. For each round, the catalytic reaction proceeded at 150 °C for 1 h. After 10 rounds, the selectivity of methanol kept almost unchanged (Supplementary Fig. 14). Moreover, the HAADF-STEM image of the used Pt_1_@MIL (“used” refers to the catalyst after 10 rounds) showed that Pt atoms were still atomically dispersed in MIL-101 without the formation of nanoparticles (Supplementary Fig. 15). In addition, only 2.6% of Pt species in Pt_1_@MIL was leached after 10 rounds as revealed by ICP-AES. To further investigate the potential industrial application, we tested Pt_1_@MIL for successive in-situ cycles of reaction. For each cycle, the catalytic reaction proceeded under 32 bar of CO_2_/H_2_ mixed gas (CO_2_/H_2_ = 1:3) at 150 °C for 6 h without the removal of catalysts from the reactor during the whole test. After 16 in-situ cycles (96 h in total), Pt_1_@MIL led to the generation of about 14.6 mmol of methanol and 1.4 mmol of formic acid in total, exhibiting high long-term stability (Fig. 2d). Accordingly, Pt_1_@MIL was highly stable during CO_2_ hydrogenation, thereby exhibiting potential industrial application.

### Formation of hydroxyl groups in Pt_1_@MIL under H_2_

**Fig. 3:**
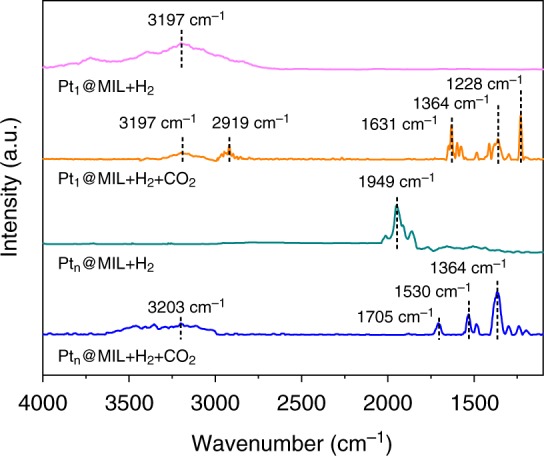
Adsorption properties of reactants on Pt@MIL. In-situ DRIFTS spectra of Pt_1_@MIL and Pt_n_@MIL after the treatment with H_2_, as well as H_2_ and CO_2_ in sequence at 150 °C

We conducted DFT calculations to investigate the hydrogen dissociation on Pt_1_@MIL. Based on DFT calculations, the whole reaction path is divided into three elementary steps that include the dissociation of H_2_ on Pt (step i), the formation of the first hydroxyl (step ii), and the formation of the second hydroxyl (step iii) (Supplementary Table 2 and Supplementary Fig. 16). Specially, H_2_ is firstly dissociated on Pt to form Pt–H bonds. Afterwards, the two dissociated H atoms on Pt atoms stepwise migrates to the dangling O_2_ moiety, resulting in the formation of two hydroxyl groups. Among these steps, step ii exhibits the highest energy barrier with the value of 0.79 eV, indicating that the formation of hydroxyl groups are able to proceed at 150 °C. To support this point, we carried out in-situ diffuse reflectance infrared Fourier transform spectroscopy (DRIFTS) measurements. Figure 3 shows the in-situ DRIFTS spectra of Pt_1_@MIL and Pt_n_@MIL after the treatment with H_2_ at 150 °C for 0.5 h. As for Pt_1_@MIL, a peak at 3197 cm^−1^ appeared, corresponding to the stretching vibration of O–H. The observation of O–H and absence of Pt–H are consistent with the DFT results. With regard to Pt_n_@MIL, the peak at 1949 cm^−1^ was assigned to the stretching vibration of Pt–H. Therefore, Pt single atom activates its coordinated O atoms to adsorb the dissociated H atoms with the formation of two hydroxyl groups. As for Pt_n_@MIL, Pt nanoparticles directly dissociate H_2_ to form Pt–H species.

To investigate the role played by H atoms in hydroxyl groups, we have explored the kinetic isotope effect (KIE) with the use of D_2_ in catalytic tests. When the reaction proceeded under CO_2_/D_2_ mixed gas (CO_2_:D_2_ = 1:3) at 150 °C, the TOF numbers of Pt_1_@MIL and Pt_n_@MIL were 21 h^−1^ and 13 h^−1^, respectively (Supplementary Fig. 17a–b). The KIE value refers to the ratio of the TOF using H_2_ as the reactant gas to that using D_2_ (*k*_H_/*k*_D_). As shown in Supplementary Fig. 17c, the KIE value of Pt_1_@MIL was 5.6 much higher than that (1.6) of Pt_n_@MIL. As such, the bond cleavage of O-D rather than Pt-D was involved in the reaction over Pt_1_@MIL.

### CO_2_ hydrogenation paths

The different adsorption properties of H atoms on Pt_1_@MIL and Pt_n_@MIL induced the variation of reaction paths in CO_2_ hydrogenation. To investigate the reaction path, we calculated the addition of the first H atom into CO_2_. For Pt_1_@MIL, the energy of HCOO* is 0.26 eV lower than that of COOH* (Supplementary Fig. 18a–b and Tables 3–4). In this case, the first H atom adds to the C atom in CO_2_ to form HCOO* as the stable intermediate on Pt_1_@MIL. In comparison, Pt_n_@MIL favors the generation of COOH* whose energy is 0.34 eV lower than that of HCOO* (Supplementary Fig. 18c–d and Tables 3–4). As such, the transformation of CO_2_ into COOH* serves as the dominating reaction channel on Pt_n_@MIL. To support this point, we conducted in-situ DRIFTS measurements of Pt_1_@MIL and Pt_n_@MIL after the treatment with H_2_ and CO_2_ in sequence at 150 °C. For Pt_1_@MIL, the peak for O–H at 3197 cm^−1^ was still observed, but its intensity was weakened relative to the case when the sample was only treated with H_2_ (Fig. 3). The peak at 1364 cm^−1^ was observed, indicating the formation of CO_2_^δ-^ species^4,18^. Moreover, the peaks at 2919, 1631, and 1228 cm^−1^ also emerged, which were assigned to the stretching vibration of C–H, the asymmetrical and symmetrical stretching vibrations of the bidentate O–C–O in HCOO* species, respectively (Fig. 3). As for Pt_n_@MIL treated with H_2_ and CO_2_ in sequence relative to the one solely exposed to H_2_, the peak for Pt–H disappeared. Moreover, besides the peak for CO_2_^δ-^, the peaks at 3203, 1705, and 1530 cm^−1^ appeared, corresponding to the stretching vibration of O–H, the stretching vibration of C=O, and the bending vibration of C–O in COOH* species, respectively (Fig. 3). The in-situ DRIFTS results indicate that HCOO* and COOH* served as the intermediates for Pt_1_@MIL and Pt_n_@MIL, respectively, consistent with the theoretical results.

**Fig. 4:**
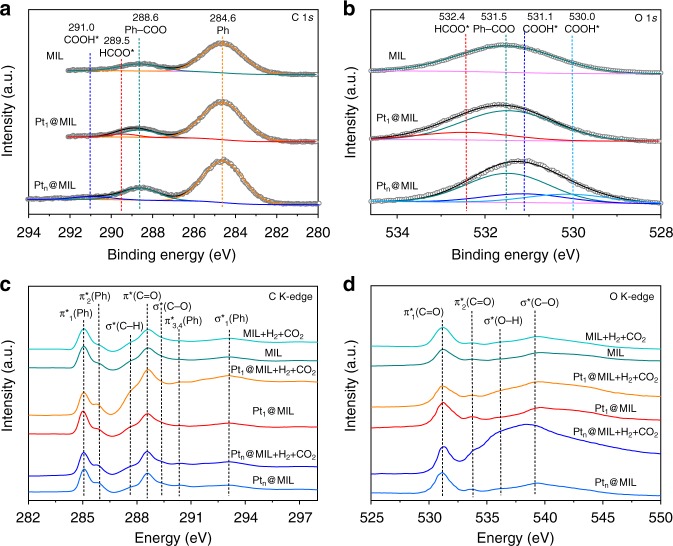
Mechanistic insight into remarkable activity of Pt_1_@MIL. **a**, **b** Quasi-situ XPS spectra of C 1 *s* and O 1 *s* for MIL-101, Pt_1_@MIL, and Pt_n_@MIL after the treatment with H_2_ and CO_2_ in sequence at 150 °C, respectively. **c**, **d** C and O K-edge XANES spectra for MIL-101, Pt_1_@MIL, and Pt_n_@MIL before/after the treatment with H_2_ and CO_2_ in sequence at 150 °C, respectively

The alteration of intermediates in CO_2_ hydrogenation over Pt_1_@MIL and Pt_n_@MIL was further supported by quasi-situ XPS. Quasi-situ XPS measurements were conducted after the treatment of the samples with H_2_ and CO_2_ in sequence at 150 °C in a reaction cell attached to the XPS end-station. As shown in Fig. 4a, the C 1 *s* spectra exhibited four typical peaks at 284.6, 288.6, 289.5, and 291.0 eV, corresponding to benzene ring in MIL-101 (Ph), carboxyl group in MIL-101 (Ph-COO), HCOO*, and COOH*, respectively^18,34–36^. In addition, different species could also be distinguished in O 1*s* spectra. Specifically, the peaks at 531.5 and 532.4 eV derived from the oxygen atoms in Ph–COO and HCOO*, respectively (Fig. 4b). Two peaks at 530.0 and 531.1 eV were assigned to the carbonyl and hydroxyl species in COOH*, respectively (Fig. 4b)^34–36^. As indicated by quasi-situ XPS spectra, HCOO* or COOH* was not observed for MIL-101. Therefore, the intermediates for Pt_1_@MIL and Pt_n_@MIL were determined as HCOO* and COOH*, respectively, consistent with in-situ DRIFTS results.

The intrinsic difference between HCOO* and COOH* lies in the exclusive existence of C–H bond in HCOO* and O–H bond in COOH*, which could be verified by C and O K-edge XANES measurements. Figure 4c shows the C K-edge XANES profiles of Pt_1_@MIL and Pt_n_@MIL before/after the treatment with H_2_ and CO_2_ in sequence at 150 °C. Before gas treatment, both Pt_1_@MIL and Pt_n_@MIL revealed the same XANES profiles which exhibited seven characteristic features. In detail, three features at 285.0, 285.9, and 290.3 eV were assigned to the excitations of the C=C π antibonding orbital for benzene ring in MIL-101 (π^*^(Ph))^37^. Besides, the peaks at 287.6, 288.5, 289.4, and 293.1 eV corresponded to the excitations of the C–H antibonding orbital (σ^*^(C–H)), the C = O π antibonding orbital (π^*^(C=O)), the C–O antibonding orbital (σ^*^(C–O)), and the σ antibonding orbital for benzene ring in MIL-101 (σ^*^(Ph)), respectively^37^. After the treatment of MIL-101 with H_2_ and CO_2_ in sequence at 150 °C, the features were almost the same as the corresponding ones before the treatment. After the sequential gas treatment, the profiles of Pt_1_@MIL and Pt_n_@MIL both showed stronger feature of π^*^(C=O) relative to that without gas treatment, indicating the transformation of CO_2_. Moreover, the feature of σ^*^(C–H) became stronger for Pt_1_@MIL, but remained almost unchanged for Pt_n_@MIL. As such, only the species generated over Pt_1_@MIL contained C–H bond. As for O K-edge XANES, the features at 531.1 and 533.7 eV corresponded to the excitations of the π^*^(C=O), while the features at 536.1 and 539.1 eV were assigned to the O–H antibonding orbital (σ^*^(O–H)) and the C–O antibonding orbital (σ^*^(C–O)), respectively^38^ (Fig. 4d). After the exposure to H_2_ and CO_2_ in sequence at 150 °C, the feature of σ^*^(O–H) became significantly stronger for Pt_n_@MIL, but stayed almost the same for Pt_1_@MIL. Accordingly, only Pt_n_@MIL induced the generation of species containing O–H groups.

In Pt_1_@MIL, every Pt single atom and its coordinated O atoms composed an active center. The metal-ligand cooperativity leads to the dissociation of H_2_ to form hydroxyl groups, wherein hydroxy H atoms added into CO_2_ to produce HCOO* as the intermediates. With regards to Pt_n_@MIL, Pt hydrides formed, offering H atoms to hydrogenate CO_2_ into COOH* as the intermediates. In general, HCOO* is reported to be hydrogenated into HCOOH* and finally converted into methanol^6,9^. By comparison, besides forming HCOOH*, COOH* is also apt to lose the hydroxyl species to generate CO* species^6,18^. CO* can be either directly desorbed to form gaseous CO, or further hydrogenated into methane^6^. Thus, experiencing the reaction path via COOH* intermediate over Pt_n_@MIL leads to the formation of gaseous products.

## Discussion

In conclusion, the metal-ligand cooperativity in Pt_1_@MIL was found to induce distinct reaction path and improve selective hydrogenation of CO_2_ into methanol relative to Pt_n_@MIL. According to mechanistic studies, CO_2_ was hydrogenated into HCOO* as the intermediate for Pt_1_@MIL, whereas COOH* served as the intermediate for Pt_n_@MIL. The unique reaction path over Pt_1_@MIL both lowered the activation energy for the enhanced activity and led to the high selectivity for methanol. Upgrading the catalysts from nanocrystals to single atoms not only enhances the atomic utilization efficiency, but also alters the catalytic mechanisms such as the adsorption of reactants or intermediates on catalysts and the reaction path. This strategy offers a powerful means to improve the catalytic performance for CO_2_ hydrogenation, and extends our understanding of single-atom catalysis.

## Methods

### Synthesis of MIL-101

The synthesis of MIL-101 followed a reported method^28^. Specifically, 2 g of Cr(NO_3_)_3_·9H_2_O, 830 mg of terephthalic acid, and 0.225 mL of HF aqueous solution (40.0% in mass fraction) were added into 25 mL of water in a 50-mL beaker, followed by stirring at room temperature for 15 min. Then, the solution was transferred into a 50-mL Teflon-lined autoclave and heated at 220 °C for 8 h. After the solution was cooled to room temperature, the product was collected by centrifugation, washed three times with water. For further purification, the solid product was sequentially stirred in 200 mL of ethanol solution (95% ethanol with 5% H_2_O) at 80 °C for 24 h, 300 mL of NH_4_F aqueous solution (30 mM) at 70 °C for 24 h, and 200 mL of water at 90 °C for 3 h. The product was collected by centrifugation, and then dried at 60 °C under vacuum.

### Synthesis of Pt@MIL

In a typical synthesis of Pt_1_@MIL, 100 mg of MIL-101 were dispersed in 60 mL of water in a 250-mL flask under magnetic stirring for 10 min. 10.1 mL of K_2_PtCl_4_ aqueous solution (0.1 mM) and 10.1 mL of NaBH_4_ aqueous solution (0.1 mM) were added into the flask through a two-channel syringe pump at a rate of 2 mL h^−1^ under magnetic stirring at room temperature. The sample was washed three times with water, and dried at 60 °C under vacuum. Further ICP result determined that the mass loading of Pt was 0.2%. The synthetic procedure for Pt_n_@MIL with the Pt loadings of 1.0% was similar to that for Pt_1_@MIL, except for increasing the amounts of K_2_PtCl_4_ and NaBH_4_. For Pt_n_@MIL, 10.4 mL of K_2_PtCl_4_ aqueous solution (0.5 mM) and 10.4 mL of NaBH_4_ aqueous solution (0.5 mM) were added into the flask through a two-channel syringe pump at a rate of 10 mL h^−1^ under magnetic stirring at room temperature. The synthesis of Pt nanoparticles with an average size of 1.2 nm was similar to that of Pt_n_@MIL except for the concentration of precursors. Specially, 100 mg of MIL-101 were dispersed in 60 mL of water in a 250-mL flask under magnetic stirring for 10 min. 10.4 mL of K_2_PtCl_4_ aqueous solution (0.3 mM) and 10.1 mL of NaBH_4_ aqueous solution (0.5 mM) were added into the flask through a two-channel syringe pump at a rate of 10 mL h^−1^ under magnetic stirring at room temperature.

### XAFS measurements

The XAFS measurements were carried out at Pt *L*_3_-edge (11564 eV) on the BL14W1 beamline^39^ of Shanghai Synchrotron Radiation Facility. The XAFS data of Pt@MIL were recorded in fluorescence mode by using a Ge solid-state detector. The calibration of the energy was based on the absorption edge of pure Pt foil. The data extraction and fitting were carried out by using Athena and Artemis codes. As for XANES, the normalized absorption refers to the experimental absorption coefficients vs. energies *μ*(*E*) after background subtraction and normalization procedures. As for EXAFS, the first-shell approximation was adopted to analyze the Fourier transformed data in *R* space of the Pt–O shell, while metallic Pt model was used to analyze that of the Pt–Pt shell. To determine the passive electron factors (*S*_0_^2^), the experimental Pt foil data were fitted while the *CN* of Pt–Pt was fixed as 12. Afterwards, *S*_0_^2^ was fixed for further analysis. The parameters such as bond distance (*R*), *CN*, and Debye Waller (*D.W*.) factor around the absorbed atoms were variable during the fitting process.

### DFT calculations

Density functional theory (DFT) calculations were performed using Gaussian program^40^ to explain the interaction mechanism between CO_2_ and Pt_1_@MIL, a cluster with 30 O atoms, 48 C atoms, 3 Cr atoms, 32 H atoms, and 1 Pt atom was adopted to simulate Pt_1_@MIL. The six carboxylate terminals on the top of benzene ring were saturated by hydrogen atoms (COO^−^ → COOH) and the twelve oxygen atoms were fixed at their crystal positions during geometric optimization. We made calculations with B3LYP-D3 functional^41^ implemented in Gaussian with the combination of the Grimme’s third generation dispersion correction (D3)^42^. For structural optimization, the standard Gaussian-type basis sets 6–31 G(d)^43^ was used for C, O, and H atoms and LANL2DZ^44^ sets for metal atoms. As for the calculation of reaction paths, we used the optimized structure for a higher-precision single-point energy calculation. In the level, the basis sets for describing C, O, and H atoms was replaced by 6–311++G(d, p)^45^, whereas the basis sets for metal atoms still adopted LANL2DZ. In order to simulate the periodic Pt (111), we used Vienna ab initio simulation package (VASP)^46,47^. The projector augmented wave (PAW) method was used to describe the interaction between ions and electrons^48^. The nonlocal exchange correlation energy was evaluated using the Perdew–Burke–Ernzerhof functional^49^. A plane wave basis set with a cutoff energy of 400 eV and a 1 × 1 × 1 *k*-point grid generated by the Monkhorst–Pack method were used to describe the Brillouin zone for geometric optimization. The atomic structures were relaxed using either the conjugate gradient algorithm or the quasi-Newton scheme as implemented in the VASP code until the forces on all unconstrained atoms were ≤0.02 eV Å^−1^.

### Catalytic tests of Pt@MIL catalysts in CO_2_ hydrogenation

The hydrogenation of CO_2_ was conducted in a 100-mL slurry reactor (Parr Instrument Company). In a typical catalytic test, the reactor was charged and discharged with 32 bar of mixed gas (CO_2_:H_2_ = 1:3) at room temperature for three times, after the addition of 10 mL of DMF and certain amounts of catalysts into the Teflon inlet. For a catalytic test, the weights of Pt_1_@MIL and Pt_n_@MIL were controlled at 500 and 240 mg, respectively, so as to keep the same amount (1.0 mg) of exposed Pt in each catalyst. The reaction proceeded under stirring with a rate of 300 rpm and 32 bar of mixed gas (CO_2_:H_2_ = 1:3) at 150 °C. After the completion of the reaction, the gaseous products were determined by GC-FID and GC-TCD. The reaction mixture in liquid phase was collected by centrifugation at 11,180 × *g* for 2 min. The test solution contained 50 μL of chloroform, as an internal standard, and 1 mL of the reaction mixture. 100 μL of the test solution was dissolved in 0.4 mL of DMSO-d_6_ for the measurements of ^1^H NMR. For each data point, the catalytic tests were repeated thrice. For the stability of Pt_1_@MIL, we applied two methods including successive reaction rounds and in-situ cycles. The addition of catalysts, solution, and reaction gases followed the same procedure as a typical catalytic test. Successive reaction rounds were carried out with the collection of catalysts for each round. Specially, after the proceeding of reaction at 150 °C for 1 h, both gaseous and liquid products were detected. Meanwhile, the catalysts were collected via centrifugation and washed thrice with DMF, followed by being re-added to the slurry reactor for the next round. In-situ cycles were carried out without opening the slurry reactor to collect the catalysts. For each cycle, after the proceeding of reaction at 150 °C for 6 h, both gaseous and liquid products were taken through tubes for detection. The TOF was calculated based on the equations.

1 $${\mathrm{TOF}} = n_{{\mathrm{CO2}}}/\left( {t \times n_{{\mathrm{surface}}\;{\mathrm{Pt}}\;{\mathrm{atoms}}}} \right) = n_{{\mathrm{CO2}}} \times \mu _{{\mathrm{Pt}}}/\left( {t \times m_{{\mathrm{surface}}\;{\mathrm{Pt}}\;{\mathrm{atoms}}}} \right)$$

2 $$m_{{\mathrm{surface}}\;{\mathrm{Pt}}} = m_{{\mathrm{cat}}} \times w \times r_{{\mathrm{surface}}}$$

In this equation, *n*_CO2_ represents the mole of converted CO_2_ molecules. *t* is the reaction time. *n*_surface Pt atoms_ represents the mole of surface Pt atoms. *μ*_Pt_ is the weight of one mole of Pt atoms. *m*_surface_ is the weight of surface Pt species in the catalysts. *m*_cat_ is the weight of catalysts. *w* represents the mass loading of Pt species. *r*_surface_ represents the ratio of surface Pt atoms to total Pt atoms. For Pt_1_@MIL, we assumed that all the Pt atoms were exposed on the surface, so that *r*_surface_ is 100.0%. For Pt_n_@MIL, *r*_surface_ was determined as 41.5% via CO pulse chemisorptions

### In-situ DRIFTS tests

The instrument for in-situ DRIFTS experiments was composed of an elevated-pressure cell (DiffusIR Accessory PN 041-10XX) and a Fourier transform infrared spectrometer (TENSOR II Sample Compartment RT-DLaTGS). The resolution of wavenumber was 4 cm^−1^ at 150 °C. The background spectrum was obtained after a N_2_ flow (1 bar) for 0.5 h at 150 °C. Then, 1 bar of H_2_ was allowed to flow into the cell at the rate of 30 sccm at 150 °C for 0.5 h, followed by flowing with 1 bar of N_2_ at the rate of 30 sccm at 150 °C for 0.5 h. To detect the species generated after the treatment of the samples with H_2_ and CO_2_ in sequence, 1 bar of H_2_ was allowed to flow into the cell at the rate of 30 sccm at 150 °C for 0.5 h, followed by flowing with 1 bar of N_2_ at the rate of 30 sccm at 150 °C for 0.5 h. Then, 1 bar of CO_2_ was allowed to flow into the cell at 25 °C for 30 min, followed by purging with 1 bar of N_2_ at 25 °C for 30 min.

### Quasi-situ XPS measurements

Quasi-situ XPS measurements were performed at the photoemission end-station on the BL10B beamline of National Synchrotron Radiation Laboratory (NSRL, China). The samples were exposed to 1 bar of H_2_ and CO_2_ in sequence at 150 °C for 0.5 h. Afterwards, the samples were moved to the analysis chamber for further XPS analysis.

### Instrumentations

TEM, HAADF–STEM, and STEM–EDX images were collected on a JEOL ARM-200F field-emission transmission electron microscope operating at 200 kV accelerating voltage. ICP-AES (Atomscan Advantage, Thermo Jarrell Ash, USA) was used to determine the concentration of metal species. NMR spectra were recorded on a Brucker-400 MHz spectrometer. The C and O K-edge X-ray absorption spectra were measured at beamline B12b of National Synchrotron Radiation Laboratory (NSRL, China) in the total electron yield (TEY) mode by collecting the sample drain current under a vacuum better than 1 × 10^–7^ Pa.

## Supplementary information

Supplementary Information

## Data Availability

The data that support the plots within this paper and other findings of this study are available from the corresponding author upon reasonable request.
