# Supplementary material for: Optimizing reaction paths for methanol synthesis from CO2 hydrogenation via metal-ligand cooperativity
Source: Nat Commun. 2019 Apr 23;10:1885. doi: 10.1038/s41467-019-09918-z (PMC6478740; doi:10.1038/s41467-019-09918-z)
Supplement: Supplementary file 1 — Supplementary Information [file 41467_2019_9918_MOESM1_ESM.pdf]

Supplementary Information for

Optimizing Reaction Paths for Methanol Synthesis from CO_2 Hydrogenation via Metal-ligand Cooperativity

Yizhen Chen[1], Hongliang Li[1], Wanghui Zhao[1], Wenbo Zhang[1], Jiawei Li[1], Wei Li[2], Xusheng Zheng[1], Wensheng Yan[1], Wenhua Zhang[1], Junfa Zhu[1], Rui Si[2] & Jie Zeng[1]

[1]Hefei National Laboratory for Physical Sciences at the Microscale, Key Laboratory of Strongly-Coupled Quantum Matter Physics of Chinese Academy of Sciences, National Synchrotron Radiation Laboratory, Key Laboratory of Surface and Interface Chemistry and Energy Catalysis of Anhui Higher Education Institutes, Department of Chemical Physics, University of Science and Technology of China, Hefei, Anhui 230026, P. R. China. [2]Shanghai Synchrotron Radiation Facility, Shanghai Institute of Applied Physics, Chinese Academy of Sciences, Shanghai 201204, P. R. China. These authors contributed equally: Yizhen Chen, Hongliang Li, Wanghui Zhao. Correspondence and requests for materials should be addressed to J.Ze. (email: zengj@ustc.edu.cn) or to R.S. (email: sirui@sinap.ac.cn)

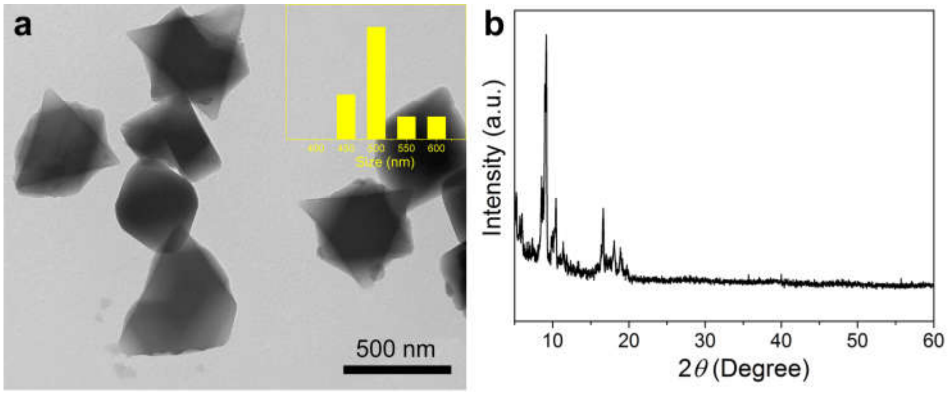

Supplementary Figure 1. (**a**) TEM images and particle size distribution of MIL-101. (**b**) XRD pattern of MIL-101.

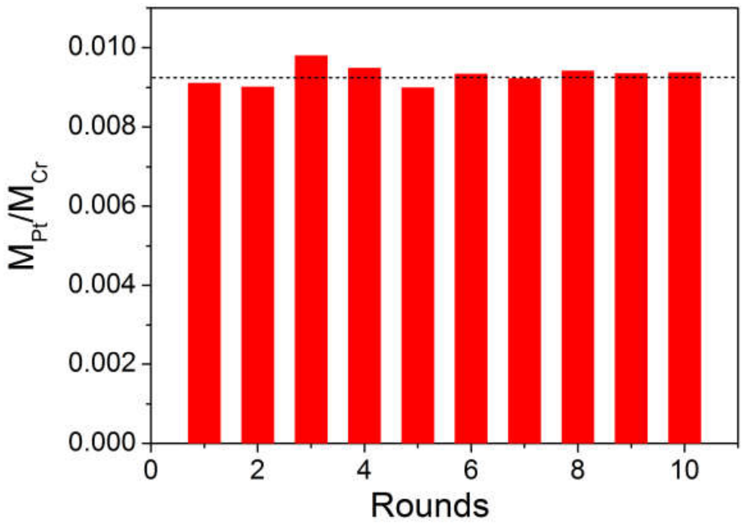

Supplementary Figure 2. The mass ratios of Pt to Cr in Pt_1@MIL after being washed for different rounds. The ratio of Pt to Cr remained almost unchanged, confirming that Pt single atoms were indeed anchored in MIL-101, rather than serving as a residual.

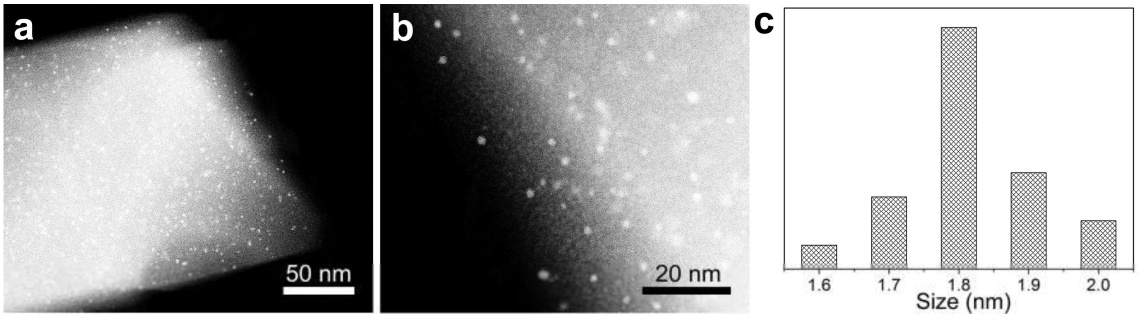

Supplementary Figure 3. (**a**, **b**) HAADF-STEM images of Pt_n@MIL. (**c**) The size distribution diagram of Pt nanocrystals in Pt_n@MIL.

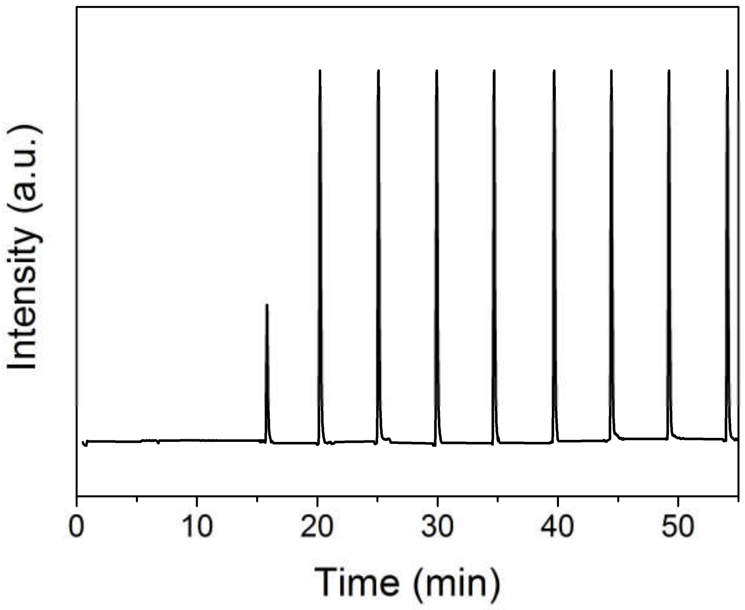

Supplementary Figure 4. CO pulse chemisorption profile of Pt_n@MIL. The ratio of surface Pt atoms to total Pt atoms in Pt_n@MIL was determined as 41.5%.

Supplementary Table 1. EXAFS data fitting results of Pt_1@MIL and Pt_n@MIL. Pt foil was used as the reference.

Sample	Pt-O		Pt-Pt		σ^2 (Å^2)	ΔE_0 (eV)
	R (Å)	*CN*	*R* (Å)	*CN*		
Pt_1@MIL	2.01±0.01	3.7±0.2	—	—	0.0030±0.0002	7.6±0.5
Pt_n@MIL	—	—	2.76±0.01	7.4±0.4	0.0050±0.0002	11.4±1.0
Pt Foil	—	—	2.76±0.01	12	0.0049±0.0002	7.5±0.5

R, distance between absorber and backscatter atoms; *CN*, coordination number; σ^2, Debye-Waller factor; ΔE_0, inner potential correction to account for the differences in the inner potential between the sample and the reference compound.

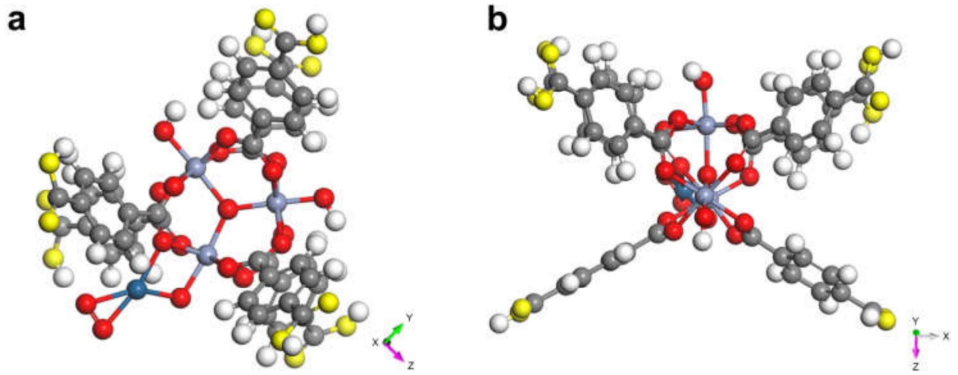

Supplementary Figure 5. (**a**, **b**) Structural models of Pt_1@MIL simulated by DFT calculations from different orientations. Blue, violet, red, gray, and white spheres represent for Pt, Cr, O, C, and H atoms, respectively. The yellow spheres represent the oxygen atoms which are fixed at their positions in crystal.

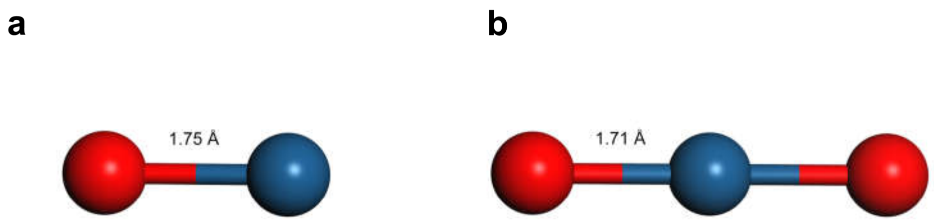

Supplementary Figure 6. (**a**, **b**) Optimized structures of Pt-O and O-Pt-O clusters. Blue and red spheres represent for Pt and O atoms, respectively.

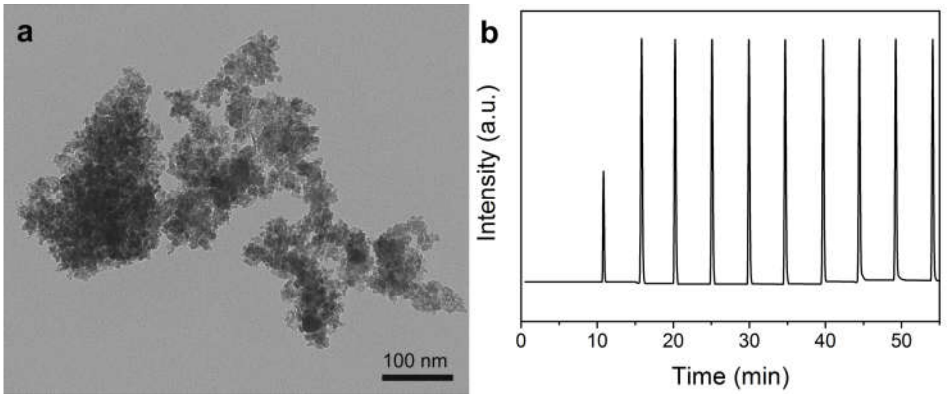

Supplementary Figure 7. (**a**) TEM image of commercial Cu/ZnO/Al_2O_3. (**b**) Profile of the adsorption and decomposition of N_2O on the surface of metallic copper for commercial Cu/ZnO/Al_2O_3. The ratio of surface Cu metal atoms to total atoms in Cu/ZnO/Al_2O_3 was determined as 32.5% by the adsorption and decomposition of N_2O on the surface of metallic copper as follows: $2Cu(s)+N_2O \rightarrow N_2+Cu_2O(s)$.

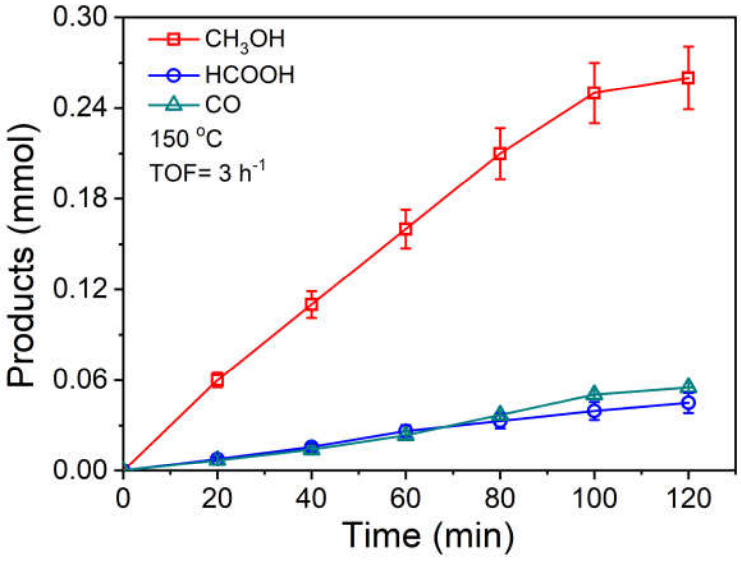

Supplementary Figure 8. Time courses of CO_2 hydrogenation catalyzed by commercial $Cu/ZnO/Al_2O_3$ in DMF under 32 bar of CO_2/H_2 mixed gas (CO_2:H_2 = 1:3) at 150 °C. For each catalytic test, the amount of $Cu/ZnO/Al_2O_3$ was controlled at 20 mg. Error bars represent standard deviation from three independent measurements.

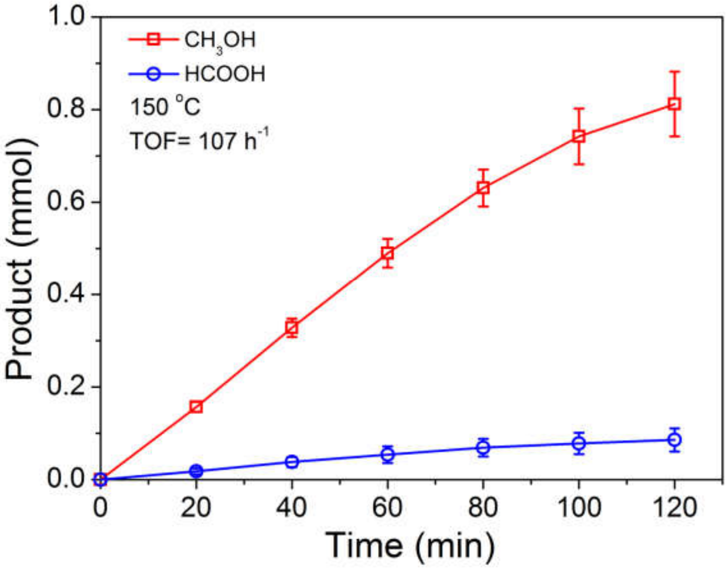

Supplementary Figure 9. (**a**) Time courses of CO_2 hydrogenation catalyzed by Pt_1@MIL in deuterated DMF (C_3D_7NO) under 32 bar of CO_2/H_2 mixed gas ($CO_2:H_2$ = 1:3) at 150 °C. The TOF number was 107 h^{-1}. Error bars represent standard deviation from three independent measurements.

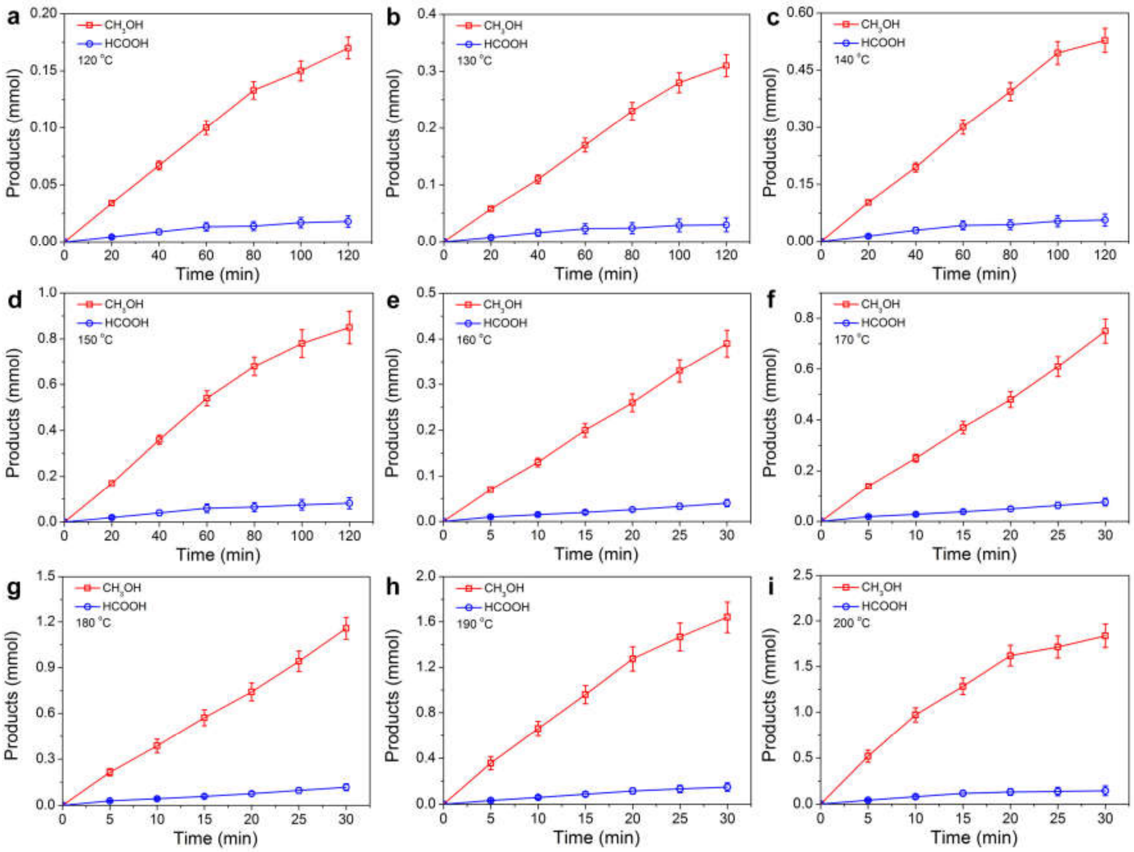

Supplementary Figure 10. Time courses of CO_2 hydrogenation catalyzed by Pt_1@MIL in DMF under 32 bar of CO_2/H_2 mixed gas ($CO_2:H_2$ = 1:3) at (**a**) 120 ^oC, (**b**) 130 ^oC, (**c**) 140 ^oC, (**d**) 150 ^oC, (**e**) 160 ^oC, (**f**) 170 ^oC, (**g**) 180 ^oC, (**h**) 190 ^oC, and (**i**) 200 ^oC. For each catalytic test, the amount of Pt_1@MIL was controlled at 500 mg to keep the amount of Pt at 1.0 mg. Error bars represent standard deviation from three independent measurements.

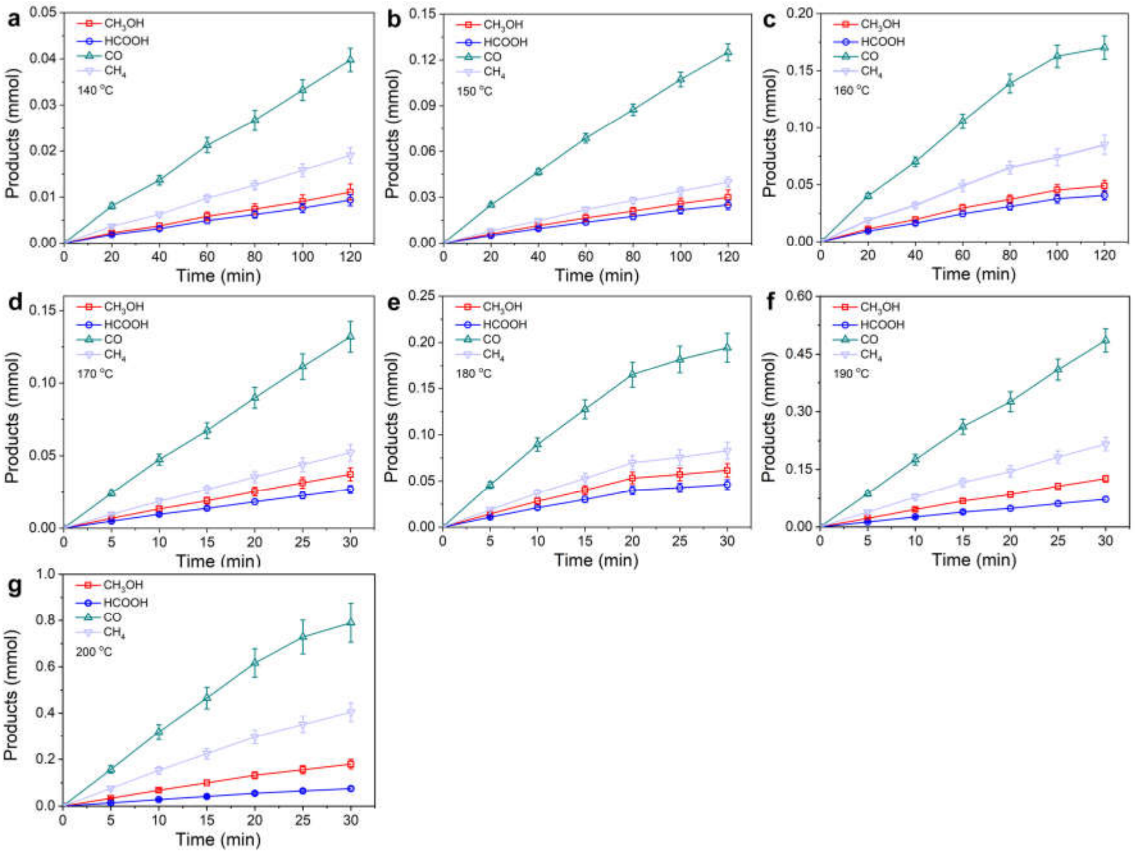

Supplementary Figure 11. Time courses of CO_2 hydrogenation catalyzed by Pt_n@MIL in DMF under 32 bar of CO_2/H_2 mixed gas (CO_2:H_2 = 1:3) at (**a**) 140 ^oC, (**b**) 150 ^oC, (**c**) 160 ^oC, (**d**) 170 ^oC, (**e**) 180 ^oC, (**f**) 190 ^oC, and (**g**) 200 ^oC. For each catalytic test, the amount of Pt_n@MIL was controlled at 240 mg to keep the same amount (1.0 mg) of surface Pt as that of Pt_1@MIL. Error bars represent standard deviation from three independent measurements.

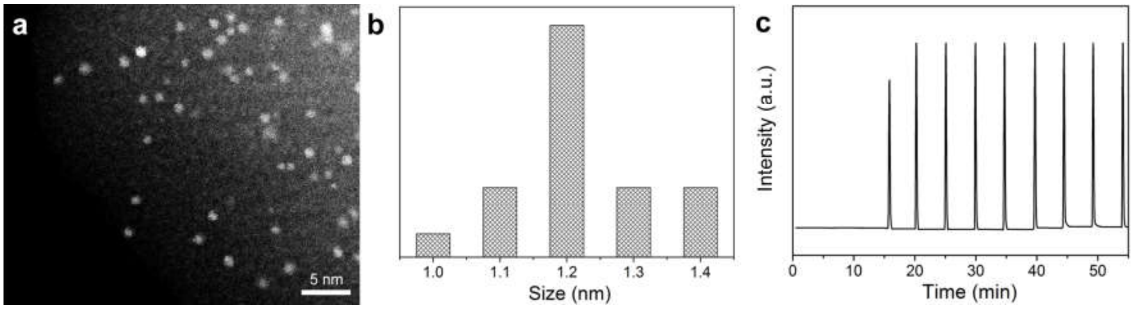

Supplementary Figure 12. (**a**) TEM image and (**b**) size distribution of 1.2-nm Pt nanoparticles on MIL-101. (**c**) CO pulse chemisorption profile of 1.2-nm Pt nanoparticles on MIL-101. The ratio of surface Pt atoms to total Pt atoms in 1.2-nm Pt nanoparticles on MIL-101 was determined as 56.4%.

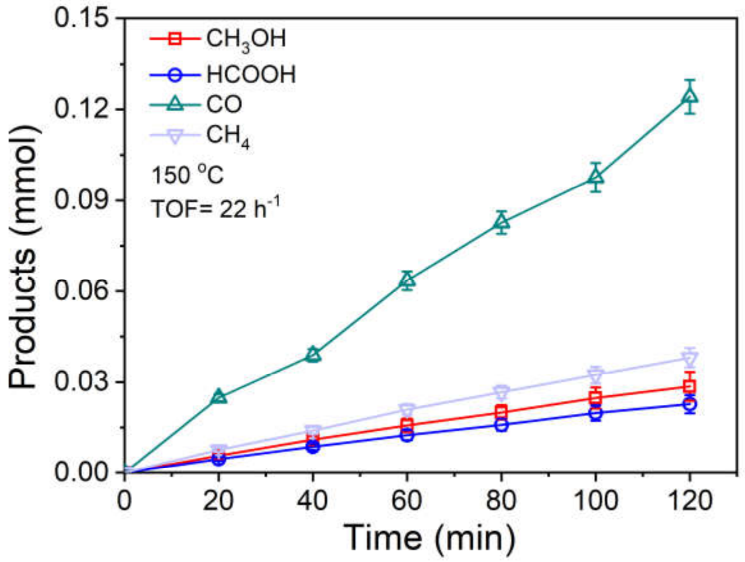

Supplementary Figure 13. Time courses of CO_2 hydrogenation catalyzed by 1.2-nm Pt nanoparticles on MIL-101 in DMF under 32 bar of CO_2/H_2 mixed gas (CO_2:H_2 = 1:3) at 150 ^oC. Error bars represent standard deviation from three independent measurements.

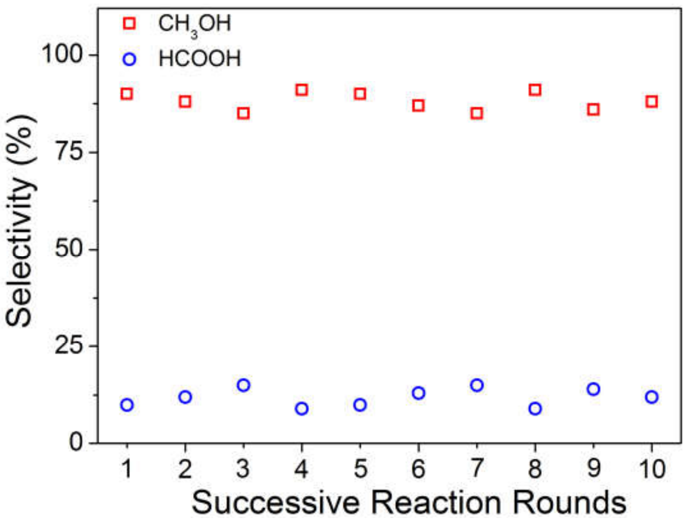

Supplementary Figure 14. Selectivity of Pt_1@MIL after conducting successive rounds of reaction. For each round, the catalytic reaction proceeded under CO_2/H_2 mixed gas ($CO_2:H_2$ = 1:3, 32 bar) at 150 ^oC for 1 h.

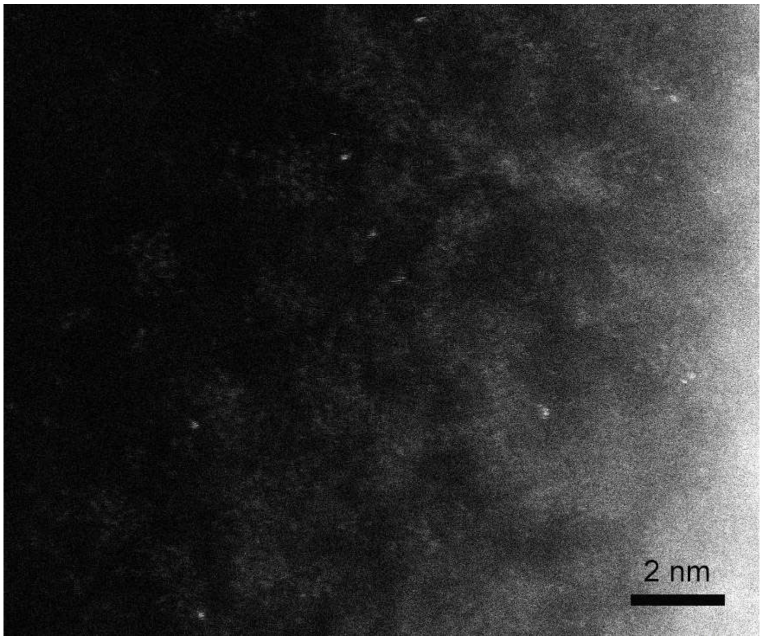

Supplementary Figure 15. HAADF-STEM image of Pt_1@MIL after ten rounds of reaction.

Supplementary Table 2. The calculated enthalpy variation ($\triangle H$) and energy barrier (E_a) of each elementary step of hydrogen dissociation on Pt_1@MIL.

	Elementary step	E_a (eV)	$\triangle H$ (eV)
step i	H_2+O_2-Pt → [2H-+O_2-]Pt	0.48	-0.25
step ii	[2H-+O_2-]Pt → [H-+OH-+O-]Pt	0.79	-0.88
step iii	[H-+OH-+O]-Pt → [2OH-]Pt	0.76	-2.38

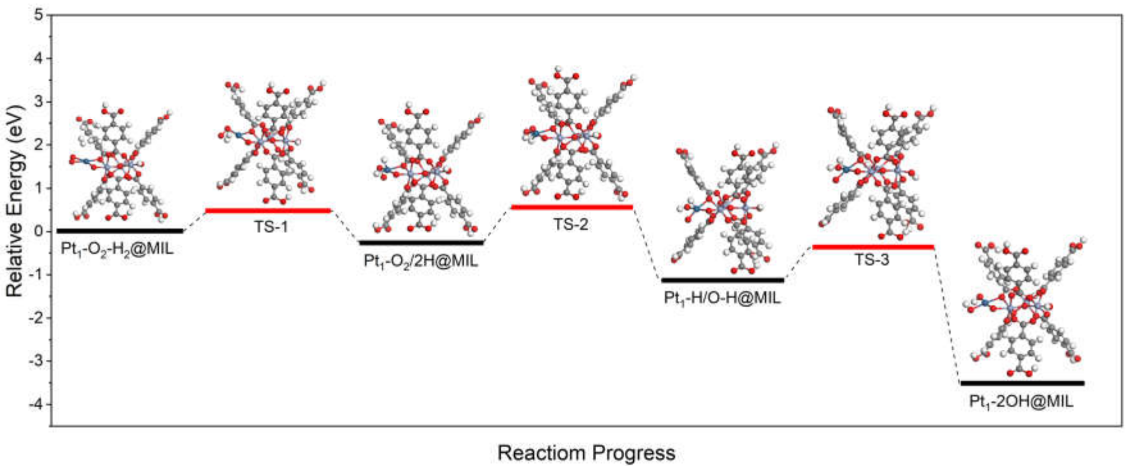

Supplementary Figure 16. Reaction paths of H_2 dissociation on Pt_1@MIL.

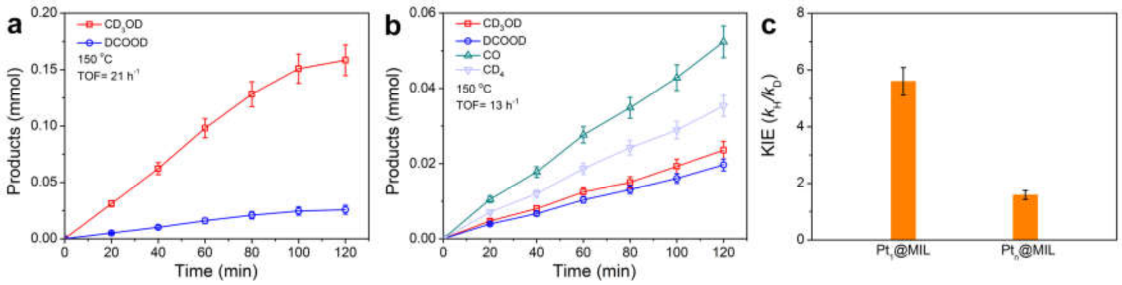

Supplementary Figure 17. Time courses of CO_2 hydrogenation catalyzed by (**a**) Pt_1@MIL and (**b**) Pt_n@MIL. The reaction was conducted under 32 bar of CO_2/D_2 mixed gas ($CO_2:D_2$ = 1:3) at 150 ^{o}C. For each catalytic test, the amounts of Pt_1@MIL and Pt_n@MIL were controlled at 500 and 240 mg, respectively, to keep the same amount (1.0 mg) of exposed Pt atoms. Error bars represent standard deviation from three independent measurements. (**c**) Comparison of KIE values of Pt_1@MIL and Pt_n@MIL.

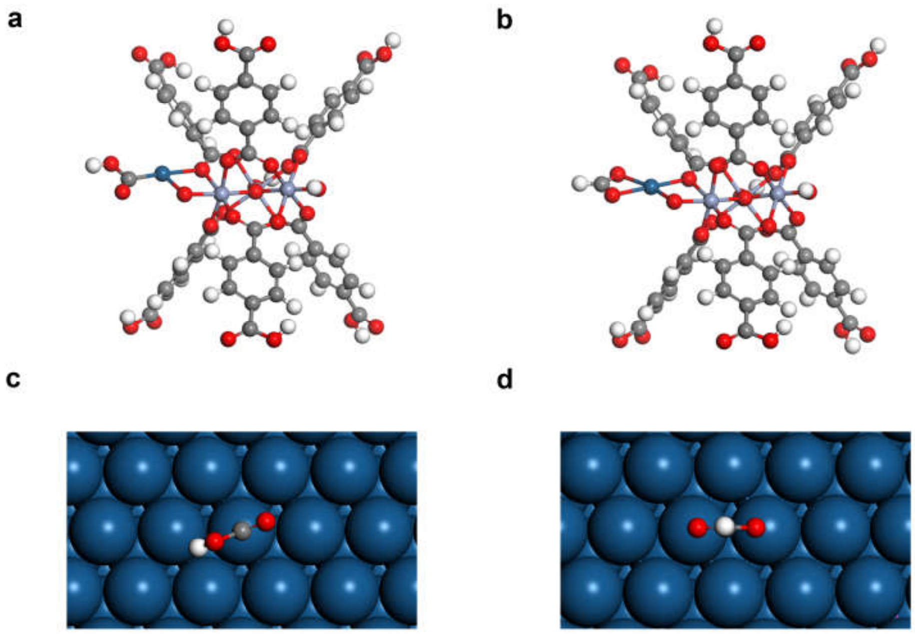

Supplementary Figure 18. (**a**, **b**) Adsorption configuration of COOH* and HCOO* on Pt_1@MIL, respectively. (**c**, **d**) Adsorption configuration of COOH* and HCOO* on Pt(111), respectively.

Supplementary Table 3. Energy of Pt_1-H_2@MIL, Pt_1-2OH@MIL, Pt_1-COOH@MIL, and Pt_1-HCOO@MIL. ΔE and ΔH represent the adsorption energy and dissociation energy of H_2 on Pt_1@MIL, respectively. E_{COOH} - E_{HCOO} represent the relative energy of the adsorbed COOH* and HCOO* on Pt_1@MIL or Pt_n@MIL.

	Pt_1-O_2-H_2@MIL	Pt_1-2OH@MIL	Pt_1-COOH@MIL	Pt_1-HCOO@MIL	Pt(111)-COOH	Pt(111)-HCOO
ΔE (eV)	-0.07	—	—	—	—	—
ΔH (eV)	—	-3.51	—	—	—	—
E_{COOH*} - E_{HCOO*}	—	—	0.26		-0.34	

Supplementary Table 4. The bond length (*L*) of the Pt single atom to the bonded O atoms.

	Pt_1-O_2@MIL	Pt_1-O_2-H_2@MIL	Pt_1-2OH@MIL	Pt_1-COOH@MIL	Pt_1-HCOO@MIL
$L_{Pt\text{-}O1}$ (Å)	1.92	1.92	1.85	1.87	1.89
$L_{Pt\text{-}O2}$ (Å)	2.08	2.08	2.15	2.23	2.06
$L_{Pt\text{-}O3}$ (Å)	2.13	2.13	1.93	——	2.15
$L_{Pt\text{-}O4}$ (Å)	2.00	2.00	1.93	——	2.08
